# Comparative transcriptomics across 14 *Drosophila* species reveals signatures of longevity

**DOI:** 10.1111/acel.12740

**Published:** 2018-04-19

**Authors:** Siming Ma, Andrei S. Avanesov, Emily Porter, Byung Cheon Lee, Marco Mariotti, Nadezhda Zemskaya, Roderic Guigo, Alexey A. Moskalev, Vadim N. Gladyshev

**Affiliations:** ^1^ Division of Genetics Department of Medicine Brigham and Women's Hospital Harvard Medical School Boston MA USA; ^2^ Genome Institute of Singapore A*STAR Singapore City Singapore; ^3^ College of Life Sciences and Biotechnology Korea University Seoul South Korea; ^4^ Bioinformatics and Genomics Program Centre for Genomic Regulation and Universitat Pompeu Fabra Barcelona Spain; ^5^ Institute of Biology Komi Science Center Russian Academy of Sciences Syktyvkar Russia; ^6^ Moscow Institute of Physics and Technology Dolgoprudny, Moscow Region Russia; ^7^ Engelhardt Institute of Molecular Biology Russian Academy of Sciences Moscow Russia; ^8^ Belozersky Institute of Physico‐Chemical Biology Moscow State University Moscow Russia

**Keywords:** aging, gene expression, lifespan, Drosophila

## Abstract

Lifespan varies dramatically among species, but the biological basis is not well understood. Previous studies in model organisms revealed the importance of nutrient sensing, mTOR, NAD/sirtuins, and insulin/IGF1 signaling in lifespan control. By studying life‐history traits and transcriptomes of 14 *Drosophila* species differing more than sixfold in lifespan, we explored expression divergence and identified genes and processes that correlate with longevity. These longevity signatures suggested that longer‐lived flies upregulate fatty acid metabolism, downregulate neuronal system development and activin signaling, and alter dynamics of RNA splicing. Interestingly, these gene expression patterns resembled those of flies under dietary restriction and several other lifespan‐extending interventions, although on the individual gene level, there was no significant overlap with genes previously reported to have lifespan‐extension effects. We experimentally tested the lifespan regulation potential of several candidate genes and found no consistent effects, suggesting that individual genes generally do not explain the observed longevity patterns. Instead, it appears that lifespan regulation across species is modulated by complex relationships at the system level represented by global gene expression.

## 1 INTRODUCTION

Since the early 20th century, fruit flies have remained a vital tool in cell biology, genetics, development, and evolution. While the best known early example is probably the use of *Drosophila melanogaster* by Thomas H. Morgan to elucidate the chromosomal theory of inheritance, other species such as *D. pseudoobscura* and *D. virilis* have long been used to study evolution and speciation (Markow & O'Grady, 2007). The entire genus *Drosophila* contains over 2,000 described species that occupy diverse ecological niches such as forests, deserts, and cosmopolitan areas (Markow & O'Grady, 2005; Schnebel & Grossfield, 1983). With the completion of full‐genome sequences of 12 *Drosophila* species (Clark et al., 2007), researchers are able to explore various aspects of their biology in much greater depth. Examination across multiple evolutionarily related lineages can reveal insights on the unique biology of flies, as well as new themes and biological mechanisms that apply across diverse life forms.

Given their relatively short life cycle, fruit flies are particularly suitable for studying longevity and aging. Under laboratory settings, the lifespan of *D. melanogaster* has been successfully increased by genetic manipulations (Clancy et al., 2001; Hwangbo, Gershman, Tu, Palmer & Tatar, 2004; Kapahi et al., 2004; Lin, Seroude & Benzer, 1998; Orr & Sohal, 1994; Parkes et al., 1998; Sun, Folk, Bradley & Tower, 2002; Tatar et al., 2001), dietary interventions (Chapman & Partridge, 1996; Grandison, Piper & Partridge, 2009; Lee et al., 2014; Magwere, Chapman & Partridge, 2004; Mair, Goymer, Pletcher & Partridge, 2003; Min & Tatar, 2006), and pharmacological treatments (Bjedov et al., 2010; Danilov et al., 2013; Wang et al., 2013). These findings are similar to those reported in other model organisms and highlight the important role of nutrient sensing, mTOR, NAD/sirtuins, insulin/IGF1 signaling pathways, and other systems in lifespan control (Fontana, Partridge & Longo, 2010; He et al., 2014).

Lifespan, weight, time to maturity, and other life‐history traits naturally differ across various *Drosophila* species as the result of millions of years of natural selection, drift, and adaptation. Since the divergence from a common ancestor, *Drosophila* lifespan has increased along certain lineages but decreased in others (Schnebel & Grossfield, 1983), indicating that longevity can be modulated in both directions on the evolutionary timescale. The heritability and stability of species lifespan across generations indicate a genetic basis for the longevity determinant(s). By identifying the genes whose expression levels correlate with lifespan across the *Drosophila* lineage, one may obtain clues about the pathways involved and ultimately the mechanisms through which nature modulates longevity. Such gene expression patterns may also be compared with known lifespan‐extension strategies to identify commonality in their effects.

Here, we used 14 *Drosophila* species spanning five taxonomical groups and examined their lifespan, body mass, development time, and gene expression profiles. We explored the relationships among various life‐history traits, identified the pathways that diverged significantly across these species, and observed the role of stabilizing selection in gene expression variation. We also identified the genes and pathways with significant positive or negative correlation to longevity (false discovery rate (FDR) < 0.05), after taking into account the influence of phylogeny and body mass differences. Finally, we analyzed our list of genes against previously published lifespan‐extension data and experimentally tested a number of candidates for longevity effects, offering various insights into regulation of longevity.

## 2 RESULTS AND DISCUSSION

### 2.1 Life‐history traits in *Drosophila*

**Figure 1:**
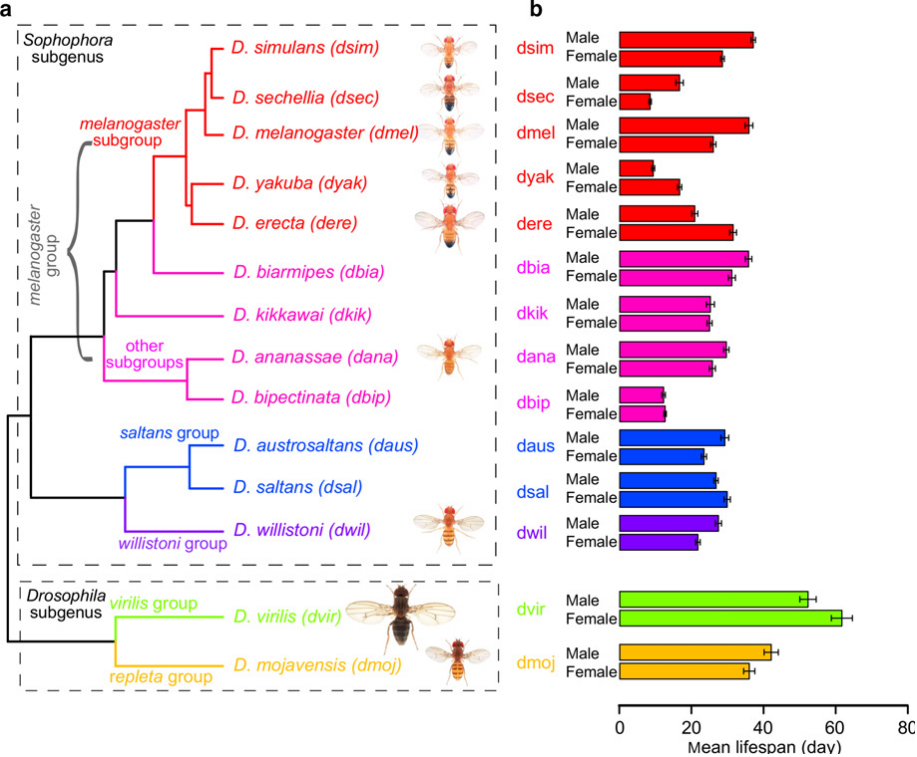
*Drosophila* species surveyed in the current study. (a) Phylogenetic relationship. The species are colored by taxonomical grouping (abbreviations of species names shown in parentheses). The tree is based on amino acid sequences of orthologs. Images of flies (all males) were obtained from Flybase (copyright: Nicolas Gompel). (b) Mean lifespan. Data are presented separately for male and female flies (Table S1). Error bars indicate 95% confidence intervals (C.I.) by Kaplan–Meier method

The 14 species surveyed in this study fall into two subgenera (*Drosophila* and *Sophophora*; Figure 1a and Table S1). Subgenus *Drosophila* is represented by *D. virilis* and *D. mojavensis* (together forming the *virilis–repleta* radiation; Markow & O'Grady, 2007), while subgenus *Sophophora* is represented by three species groups: *melanogaster* (*D. melanogaster* and eight related species), *saltans* (*D. austrosaltans* and *D. saltans*), and *willistoni* (*D. willistoni*). Those within the *melanogaster* species group can be classified further into different subgroups (Figure 1a).

We first characterized their lifespan, body mass, and developmental time, with the flies kept at 25°C. Among these species, *D. virilis* was morphologically distinct, largest in size (almost 2 mg), and longest‐lived (mean lifespan: male 52.8 days; female 62.3 days), whereas *D. sechellia*,* D. yakuba*, and *D. bipectinata* were among the shortest‐lived (mean lifespan 8–16 days) and relatively small (0.5–0.8 mg; Figure 1b, Figure S1 and Table S1). The mean lifespans of the remaining species ranged between 20 and 40 days. They are shorter than the lifespans of flies kept under 20°C (Schnebel & Grossfield, 1983; Table S1). The more rapid aging in flies under higher temperatures was also observed previously (Miquel, Lundgren, Bensch & Atlan, 1976). Within each species, the female flies were generally larger in size than the male flies (Table S1). Furthermore, we observed positive correlation between their body weights and median lifespans on log‐scale (*p*‐value = 4.66 × 10^−3^ for male and 1.17 × 10^−2^ for female), although the relationship was partly driven by *D. virilis* (excluding *D. virilis*:* p*‐value = 8.01 × 10^−3^ for male and 0.22 for female). The long‐lived *D. virilis* also had the longest developmental time (18 days), whereas the other species took ~10–12 days (Table S1).

### 2.2 Gene expression variation reflects evolutionary relationships

To measure gene expression, we collected young adult male flies of each species for whole‐body RNA extraction and sequencing. After normalization by library sizes and removal of genes expressed at low levels (Section 4), log‐RPKM values were calculated for 6,510 gene ortholog sets. Overall, the expression profiles were similar to one another, with Spearman correlation coefficients of species pairs ranging between 0.68 and 0.90. Our expression data were also consistent with the previous studies of gene expression across multiple *Drosophila* species (Chen et al., 2014; Zhang, Sturgill, Parisi, Kumar & Oliver, 2007; Figure S2a).

**Figure 2:**
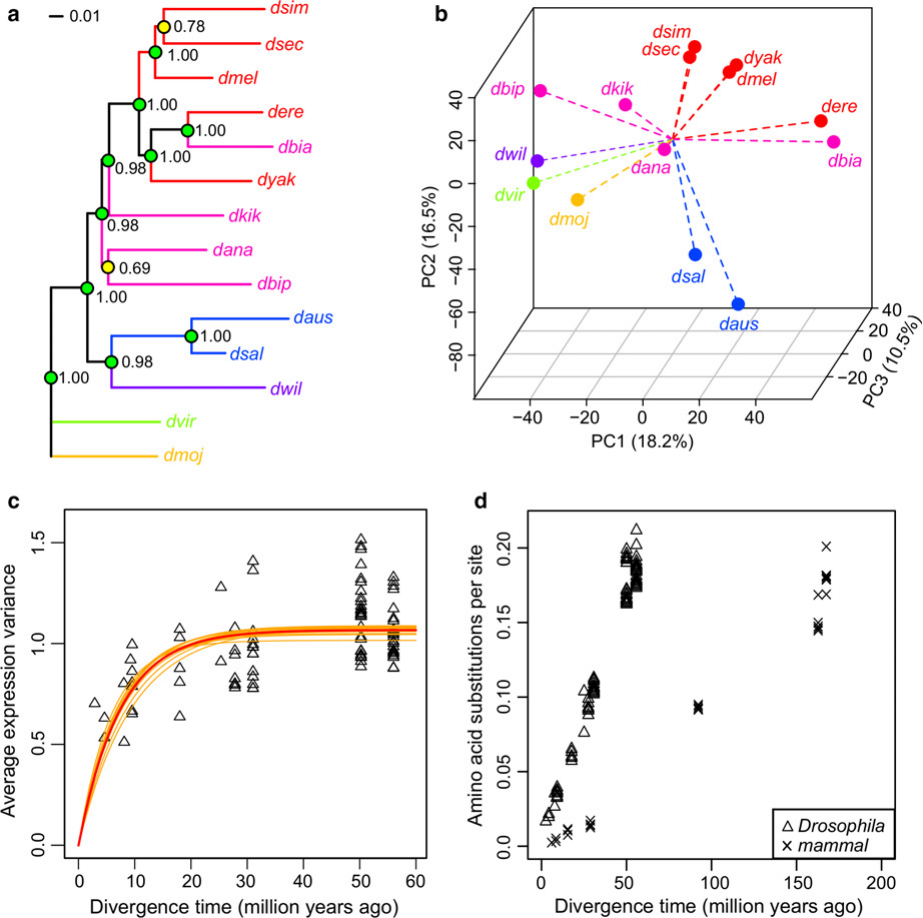
Gene expression reflects evolutionary relationships. (a) Phylogram constructed based on gene expression. *Drosophila virilis* was used as outgroup. Reliability of the branching pattern was assessed by 1,000‐time bootstrap across the genes (bootstrap values next to the nodes; green: ≥0.9; yellow: 0.6–0.9). (b) Principal component analysis. Proportion of variance explained by each principal component (PC) is indicated in parentheses. (c) Gene expression divergence reaches a plateau. Each triangle represents a pair of species. The red curve represents the best‐fit line based on the model previously described (Bedford & Hartl, 2009), with the following parameters: selection parameter α = 0.0673 (95% C.I.: 0.0639–0.0721); drift parameter σ^2^ = 0.142 (95% C.I.: 0.136–0.152; Section 4). Orange curves represent the best‐fit lines when each individual species was removed, one at a time (α ranged between 0.0530 and 0.0867). (d) Amino acid substitutions per site increase faster in *Drosophila* than in mammals. Amino acid substitutions per site between species pairs were calculated based on concatenated, gap‐free alignment of orthologs

To determine whether the evolutionary relationship of these species was reflected in their expression variation, we constructed gene expression phylograms using a distance matrix of 1 minus Spearman correlation coefficients and the neighbor‐joining method (Brawand et al., 2011; Clark et al., 2007). The resulting topology was largely consistent with their phylogeny (Figure 2a). For example, there was a clear separation between subgenera *Drosophila* and *Sophophora*; all nine species of the group *melanogaster* fell under a single clade; and the two species of the *saltans* group clustered with each other. Most of the nodes received strong support in 1,000‐time bootstrapping. However, *D. biarmipes* was placed within the subgroup *melanogaster*, which might reflect divergence between genetic distance and expression distance. Other methods also produced very similar results (Figure S2b–d).

Principal component analysis also showed that the species clustered according to their lineages (Figure 2b), with the first three principal components (PCs) accounting for ~45% of the total variance. To understand the basis of the segregation pattern, we performed pathway enrichment analysis using DAVID (Huang da, Sherman & Lempicki, 2009; Huang da, Sherman & Lempicki, 2009) on the genes with top 5% contribution to each PC (~300 genes each; Table S2, Figure S2e). In PC1, the top three functional clusters were ribosome/ribosomal proteins; ATP/nucleotide binding; and mitochondrial electron transport chain and oxidative phosphorylation (Table S2A). Separation along PC2 was largely due to differences in transcription regulation and transcription factor binding (Table S2B), whereas PC3 was related to glycine, serine, and threonine metabolism (Table S2C). In other words, these are the processes that diverge most significantly across the 14 species (without explicitly considering their lifespan differences) and may account for their phenotypic diversities. It should be noted, however, that the underlying phylogenetic structure of the data may render the PCA biased. There is currently no consensus for a multivariate phylogenetic comparative method (Uyeda, Caetano & Pennell, 2015), so the results need to be interpreted with caution.

### 2.3 Expression variation is best described by stabilizing selection

In the absence of selective pressure, the variation between a pair of species is expected to increase linearly with divergence time and can be modeled by a Brownian motion (BM) process (Felsenstein, 1985); this has been observed for transcription of many genes in mammals (especially among primates; Brawand et al., 2011; Khaitovich et al., 2004). On the other hand, previous studies based on genomes and transcriptomes observed that a large fraction of the genes in *Drosophila* were likely subjected to stabilizing selection (Bedford & Hartl, 2009; Clark et al., 2007; Kalinka et al., 2010; Rifkin, Kim & White, 2003), such that the increase in gene expression divergence eventually reaches a plateau (Bedford & Hartl, 2009) and may be better described by Ornstein‐Uhlenbeck (OU) process (Butler & King, 2004; Martins & Hansen, 1997).

**Figure 3:**
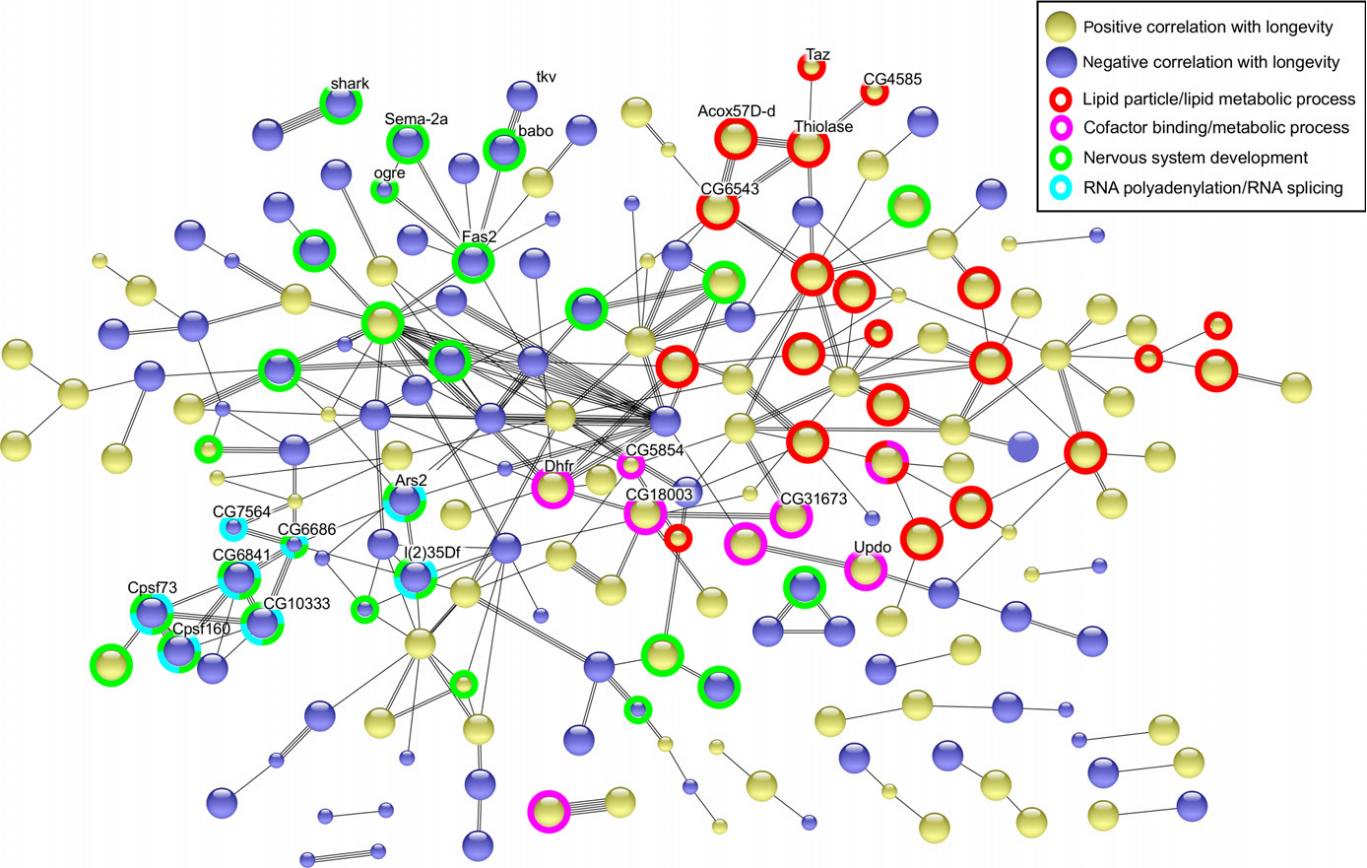
Protein–protein interaction network among the top hits correlating with median lifespan. The interaction network is based on STRING database (evidence view). Genes without interacting partners are omitted. Selected pathways are indicated by colored rings. See Figure S4 for detailed labeling

In agreement, we observed a nonlinear relationship with a plateau phase when plotting the average expression variances of the *Drosophila* species pairs against their divergence time [assuming subgenera *Sophophora* and *Drosophila* diverged at 56 Million Years Ago (Mya; Russo, Mello, Frazão & Voloch, 2013)] (Figure 2c). Fitting the data with a previously published equation (Bedford & Hartl, 2009), we confirmed the selection parameter α was significantly >0 (α = 0.0673; 95% confidence interval: 0.0639–0.0721), and the observed relationship did not depend on any particular species (Figure 3c). Data simulation also suggested the trajectory resembled the pattern produced under an OU model more than that produced under a BM model (Figure S3a). A number of other estimates on the divergence time between subgenus *Sophophora* and subgenus *Drosophila* can be found in the literature (e.g. 63 Mya, Gao, Hu, Toda, Katoh & Tamura, 2011; and 25–40 Mya, Obbard et al., 2012; 40 Mya, Russo, Takezaki & Nei, 1995). However, the same plateau feature could still be observed using these other estimates, suggesting it was underpinned mainly by the expression variances among the species pairs (i.e. the vertical axis of Figure 2c). For comparison, a similar analysis was performed using the brain and liver data of nine mammalian species (Brawand et al., 2011), but the plateau feature was not as strong (Figure S3b,c). Since *Drosophila* diverged more recently than the mammals, it was surprising to see the plateau feature in the former but not the later. Indeed, when amino acid substitutions per site between species pairs were plotted against divergence time, *Drosophila* produced a much steeper slope than the mammals (Figure 2d). It was previously reported that the substitution rate at silent sites in nuclear genes in *Drosophila* was about three times higher than that in mammals (Sharp & Li, 1989). This likely reflects the notion that the evolutionary divergence covered by the genus *Drosophila* equals or exceeds that of the entire mammalian radiation, probably due to the short generation time of fruit flies (Clark et al., 2007; Stark et al., 2007).

To examine the evolutionary models at the individual gene level, phylogenetic signals were measured using two metrics, Pagel's λ (Pagel, 1999) and Blomberg's K (Blomberg, Garland & Ives, 2003). These metrics are usually high for genes that follow BM model, but can be weakened by processes such as stabilizing selection. We found that the phylogenetic signals were low for many genes (Figure S3d; median values: λ = 0.03, *K* = 0.41), suggesting most of the variations observed were not fully accounted for by the BM model. In addition, when we compared the goodness of fit of individual gene expression under BM model against OU models with up to three optima (Butler & King, 2004; Kalinka et al., 2010), we found over 85% of the genes fitted better with one of the OU models than with the BM model (Figure S3e,f), similar to the percentage previously observed (Kalinka et al., 2010). Together, these data suggest that stabilizing selection likely plays an important role in influencing the gene expression patterns in *Drosophila*.

### 2.4 Gene expression correlating with longevity

To identify the genes that correlate with species longevity, the phylogenetic generalized least‐squares approach was employed to adjust for the evolutionary relationship (Felsenstein, 1985; Freckleton, Harvey & Pagel, 2002; Grafen, 1989; Martins & Garland, 1991). Regression was performed between expression values and male median lifespan (“ML”), different models of trait evolution were tested, and the best‐fit model was then selected based on maximal likelihood (Table S3). Given that *D. virilis* was much larger in size and longer‐lived than the other species, we also performed regression after excluding the *D. virilis* data (Section 4). We found 384 out of the 6,510 genes with significant regression slope at *p*‐value < .05, 93 of which with *p*‐value < .01. Even after excluding the data point outlier representing longest living species (*D. virilis*), ~60% of the genes remained statistically significant (i.e., *p*‐value < .05; Table S3). However, after adjusting for multiple testing, none of these correlations reached statistical significance (median FDR = 0.79; further discussed below).

Pathway enrichment analysis was performed using DAVID separately for those with positive and negative correlation. Among those with positive correlation with ML, the top terms were related to lipid synthesis and metabolism, including “oxidation–reduction process,” “lipid particle,” “biosynthesis of antibiotics,” “peroxisome” (these four pathways all had FDR < 0.05); and to a weaker extent, “fatty acid biosynthetic process” and “fatty acid metabolism” (Table S3). For negative correlation with ML, the enriched terms included “plasma membrane,” “adult behavior,” “protein tyrosine kinase activity,” “visual behavior,” and “neurogenesis,” although they did not pass FDR < 0.05 (Table S3).

In addition, we visualized protein–protein interactions among our top hits using STRING version 10.5 (Szklarczyk et al., 2017) and found the network was significantly enriched in interactions (*p*‐value = 9.75 × 10^−4^; Figures 3 and S4). We also generated 1,000 sets of random genes (each set consisting of the same number of genes as our top hits) and confirmed that the protein–protein interactions in our top hits network had FDR < 0.001.

Among the genes positively correlating with lifespan, those found in lipid metabolic processes or lipid particles clustered together (Figure 3). For example, *Thiolase*,* Acox57D‐d* (acyl‐coenzymeA oxidase at 57D distal), and *CG6543* (enoyl‐CoA hydratase) are involved in fatty acid beta‐oxidation, and two of them (*Thiolase* and *CG6543*) code for mitochondrial matrix proteins (St Pierre, Ponting, Stefancsik & McQuilton, 2014). *Taz* and *CG4585* are also involved in the metabolism of phospholipids. Several studies in *Drosophila‐*reported links between fatty acid oxidation and longevity control: Flies overexpressing genes involved in beta‐oxidation were longer‐lived and more resistant to oxidative stress induced by paraquat treatment (Lee, Lee, Paik & Min, 2012), while knockout of *Thiolase* significantly shortened lifespan (Kishita, Tsuda & Aigaki, 2012).

Another smaller cluster consisted of genes implicated in cofactor binding and metabolism of small organic molecules, including *Dhfr* (dihydrofolate reductase), *CG18003* (glycolate oxidase), *Updo* (uroporphyrinogen decarboxylase), *CG5854* (contains an NAD(P)‐binding domain), and *CG31673* (glyoxylate reductase activity).

In addition, some of the top genes were previously found to influence longevity in flies. For example, cyclin‐dependent kinase Cdk5 requires an activating subunit (p35) for its full biological function, and flies with p35 mutation had significantly shortened lifespan and age‐dependent loss of motor function (Connell‐Crowley, Vo, Luke & Giniger, 2007). Another gene was *Gclm*, which codes for the modulatory subunit of glutamate–cysteine ligase (GCL), the rate‐limiting enzyme in *de novo* glutathione biosynthesis. Global overexpression of GCLm in flies extended the mean lifespan by 24%, and neuronal overexpression of the catalytic subunit (GCLc) increased mean and maximum lifespans by up to 50% (Orr et al., 2005).

Many genes negatively correlating with lifespan were involved in nervous system development (Figure 3). Among them were several cell surface receptors and signaling molecules, such as *Fas2*, which codes for a cell adhesion protein Fasciclin 2 that interacts with semaphorin (Smad) and connectin to regulate axon fasciculation (Yu, Huang & Kolodkin, 2000); *babo*, which codes for a type I activin receptor and regulates cell proliferation by stimulating Smad2‐dependent pathways (Brummel et al., 1999); and *shark*, which codes for SH2 ankyrin repeat tyrosine‐protein kinase and is required for dorsal closure during development (Fernandez et al., 2000). Also present were component of gap junction (*ogre*) and TGF‐beta receptor (*tkv*).

Downregulation of activin signaling by forkhead transcription factor (FOXO) in muscle tissues of flies has been shown to improve muscle performance, reduce secretion of insulin peptides from brain, and extend lifespan (Bai, Kang, Hernandez & Tatar, 2013). Flies with *babo* knockdown in muscle lived about 20% longer than wild‐type, and pathway analysis of FOXO gene targets revealed enrichment of processes involved in postembryonic development, neuron differentiation, axonogenesis, and regulation of transcription and growth (Bai et al., 2013), similar to the terms we observed here (Table S3).

Another cluster included several genes affecting RNA polyadenylation (cleavage and polyadenylation specificity factor *Cpsf160* and *Cpsf73*) and RNA splicing (e.g., *CG6841*,* CG10333*,* CG6686*,* Ars2*,* CG7564*, and *l(2)35Df*; St Pierre et al., 2014). Many of them were also involved in the nervous system development, as alternative splicing has very important roles in modulating neuronal maturation and functions (Li, Lee & Black, 2007).

### 2.5 Comparison with longevity databases

To see if the longevity expression pattern we observed across the different fly species agreed with the patterns previously reported in *D. melanogaster* lifespan experiments, we compared the gene expression changes here against the published microarray experiments in flies, in which changes in longevity were induced by dietary or genetic interventions (Table 1). Treatments included dietary restriction in different strains of *D. melanogaster*, overexpression of *Sir2*, the dominant mutation *ovo* ^*D1*^ (to repress egg maturation in females), knockdown of tumor suppressor *p53*, and ablation of corpora allata (the endocrine gland that generates juvenile hormone). We checked whether the direction of correlation of our top genes was consistent with the up‐ or downregulation observed in these studies. Of the 11 microarray experiments, nine exhibited significant similarity with our signatures, with 58%–69% of the genes showing the same direction of change (binomial *p*‐value ranging between 2.92 × 10^−2^ and 4.90 × 10^−6^; Table 1). In other words, while only about two‐thirds of the genes overlapped between the cross‐species longevity signature and the lifespan alternation experiments in *D. melanogaster*, the patterns were unlikely purely due to random chance and might in fact represent certain coordinated changes during lifespan regulations.

**Table 1:** Comparison between the top hits and previously published microarray experiments

Microarray (GEO accession)	Tissue	Strain	Treatment (versus wild‐type)	Correlate with ML			
				Same direction	Opposite direction	Percentage matching	*p*‐value
GSE37537	Whole body	–	Dietary restriction	94	106	47%	8.21 × 10^−1^
GSE26724	Head/thorax	Canton‐S	Dietary restriction	61	33	**65%**	**2.54 × 10** ^**−3**^
GSE48145	Whole body	–	ovoD mutant (sterile)	145	85	**63%**	**4.60 × 10** ^**−5**^
GSE48145	Whole body	–	Corpora allata knockout (CAKO)	45	37	55%	2.20 × 10^−1^
GSE48145	Whole body	–	ovoD mutant with CAKO	146	88	**62%**	**9.07 × 10** ^**−5**^
GSE26726	Whole body	yw, w1118	Dietary restriction (10 days)	112	75	**60%**	**4.15 × 10** ^**−3**^
GSE26726	Whole body	yw, w1118	Dietary restriction (40 days)	48	29	**62%**	**1.98 × 10** ^**−2**^
GSE26726	Whole body	Canton‐S	dietary restriction (10 days)	109	79	**58%**	**1.71 × 10** ^**−2**^
GSE26726	Whole body	Canton‐S	Dietary restriction (40 days)	86	62	**58%**	**2.92 × 10** ^**−2**^
GSE26726	Whole body	yw, w1118	p53 knockdown (10 days)	43	20	**68%**	**2.58 × 10** ^**−3**^
GSE26726	Whole body	yw, w1118	sir2 overexpression (10 days)	91	40	**69%**	**4.90 × 10** ^**−6**^

The data were obtained from Gene Expression Omnibus (GEO) database. Microarray experiments were carried out in *D. melanogaster* (female) with lifespan‐extension effects. Differentially expressed genes in each microarray experiment were compared to our list of top hits to determine if they were in the same or opposite direction of correlation. *p*‐values are based on binomial distribution (*p*‐value < .05 are in bold).

In addition, we searched GenAge and GenDR databases (de Magalhães, Curado & Church, 2009; Plank, Wuttke, van Dam, Clarke & de Magalhaes, 2012) to test if any of our longevity signature genes were experimentally confirmed for longevity effects in yeast, flies, worms, and mice. Specifically, we checked whether the genes showing positive correlation in our list had pro‐longevity and those showing negative correlation had antilongevity effects. Among the ML gene list, 18 genes were found in the database and 14 of them showed the expected direction of correlation (binomial *p*‐value = .015; Table 2), although many of these effects were due to decreased lifespan. If we only considered increased lifespan in response to genetic manipulations, then the overlap was no longer significant.

**Table 2:** Top hits that were previously reported to influence lifespan in model organisms

	Fly Gene ID	Lifespan Corr.	Model Organism	Symbol	Longevity effect	Matching direction	Note	Reference
ML	FBgn0086687	Positive	Worm	fat‐7	Pro‐longevity	Same	RNAi decreased lifespan	GenAge/GenDR
	FBgn0037020	Positive	Yeast	PEX14	Necessary for Fitness	Same	Deletion decreased lifespan	GenAge/GenDR
	FBgn0025352	Positive	Fly	Thiolase	Pro‐longevity	Same	Knockout decreased lifespan	Kishita et al. (2012)
	FBgn0014010	Positive	Worm	rab‐5	Pro‐longevity	Same	RNAi decreased lifespan	GenAge/GenDR
	FBgn0039636	Positive	Yeast	ATG14	Necessary for Fitness	Same	Deletion decreased lifespan	GenAge/GenDR
	FBgn0000442	Positive	Worm	pkg‐1	Antilongevity	Opposite	Mutation increased lifespan	GenAge/GenDR
	FBgn0013762	Positive	Fly	Cdk5	Pro‐longevity	Same	Loss of function decreased lifespan	GenAge/GenDR
	FBgn0036813	Positive	Worm	atg‐3	Pro‐longevity	Same	RNAi decreased lifespan	GenAge/GenDR
	FBgn0046114	Positive	Fly	Gclm	Pro‐longevity	Same	Overexpression increased lifespan	GenAge/GenDR
	FBgn0004868	Positive	Worm	gdi‐1	Pro‐longevity	Same	RNAi decreased lifespan	GenAge/GenDR
	FBgn0011205	Positive	Worm	pnk‐1	Pro‐longevity	Same	RNAi decreased lifespan	GenAge/GenDR
	FBgn0038325	Negative	Worm	atg‐4.2	Pro‐longevity	Opposite	RNAi decreased lifespan	GenAge/GenDR
	FBgn0023076	Negative	Mouse	Clock	Pro‐longevity	Opposite	Deletion decreased lifespan	GenAge/GenDR
	FBgn0011725	Negative	Worm	ccr‐4	Pro‐longevity	Opposite	RNAi decreased lifespan	GenAge/GenDR
	FBgn0003380	Negative	Mouse	Kcna3	Antilongevity	Same	Deletion increased lifespan	GenAge/GenDR
	FBgn0011300	Negative	Fly	babo	Antilongevity	Same	RNAi increased lifespan	Bai et al. (2013)
	FBgn0020379	Negative	Yeast	RFX1	Antilongevity	Same	Deletion increased lifespan	GenAge/GenDR
	FBgn0035918	Negative	Yeast	CDC6	Antilongevity	Same	Knockout increased lifespan	GenAge/GenDR

The data were compiled primarily based on GenAge and GenDR databases and were further supplemented with literature searches. The “matching direction” column indicates whether longevity effects are in the same or opposite direction of correlation in our top list.

### 2.6 Experimental verification by *in vivo* RNAi knockdown

**Figure 4:**
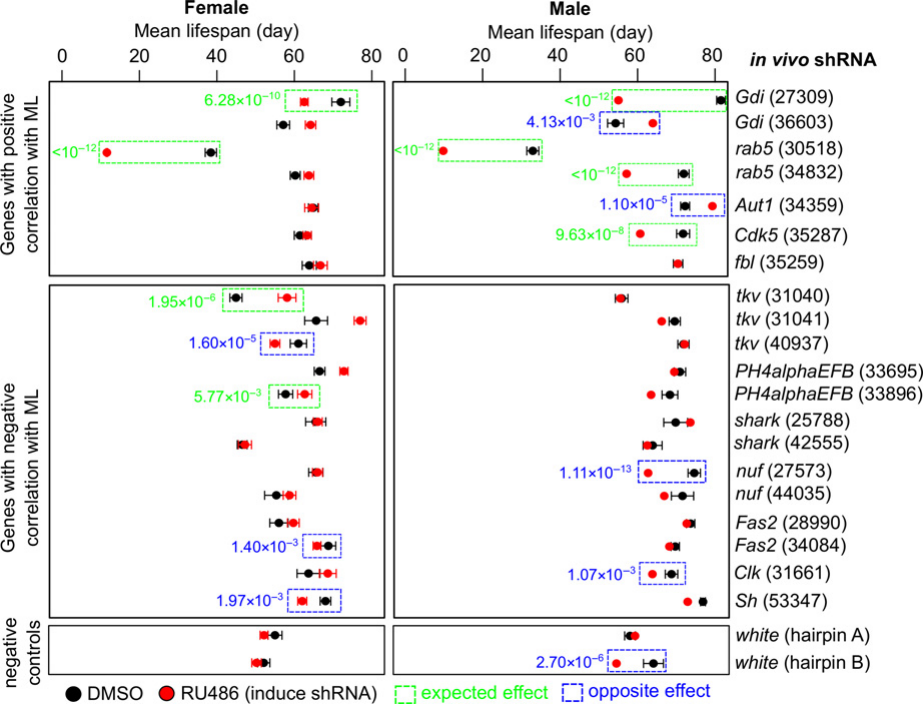
Longevity effect of RNAi knockdown of selected genes. Each gene was targeted by one or more hairpins (identified by the stock numbers of the Transgenic RNAi Project in the parentheses). Black dots and red dots refer to the mean lifespan of flies treated with vehicle or with RU486 (inducing shRNA), respectively. The error bars denote standard error. Those with significant lifespan effects (G‐rho family tests *p*‐value < .01 and hazard ratio > 1.5) are boxed in green (if the longevity effect was the expected direction) or in blue (if the longevity effect was the opposite direction) and their *p*‐values indicated. See Table S4 for more details

The Transgenic RNAi Project (TRiP) has generated and made publicly available a large number of transgenic RNAi fly stocks (Ni et al., 2008). In this resource, the transgenic shRNA is placed under UAS/GAL4 promoter and can be induced by feeding flies with the drug RU486 (Osterwalder, Yoon, White & Keshishian, 2001). To test if manipulation of individual genes identified as a part of the longevity signatures affects lifespan, we examined 20 lines representing five genes with positive correlation to longevity and seven genes with negative correlation to longevity in both male and female transgenic lines (Figure 4, Table S4). The genes were selected based on transgenic stock availability and number of individual hairpins. Two lines targeting *white* gene were included as negative control. The lifespan of each line treated with and without RU486 were measured and quantified by Kaplan–Meier statistics and hazard ratios (Harrington & Fleming, 1982; Kaplan & Meier, 1958).

Using the cutoff of G‐rho family tests *p*‐value < .01 and hazard ratio > 1.5, the overall experimental results varied depending on the gender of flies. Among the genes with positive correlation to longevity, *Gdi* hairpin 27,309 (BL stock center designations) and *rab5* hairpin 30,518 significantly reduced lifespan in both male and female flies, whereas *rab5* hairpin 34,832 and *Cdk5* hairpin 35,287 reduced lifespan in male flies only. On the other hand, *Gdi* hairpin 36,603 and *Aut1* hairpin 34,359 increased lifespan in male flies (no effect on females; Figure 4, Table S4). For the genes with negative correlation to longevity, one hairpin targeting *tkv* (hairpin 31,040) and one targeting *PH4alphaEFB* (hairpin 33,896) increased lifespan in female flies, but the effects were not observed in males. Among the remaining 20 hairpin‐gender pairs, five instances (*tkv* hairpin 40,937, *Fas2* hairpin 34,084, and *Sh* hairpin 53,347 in females; *nuf* hairpin 27,573 and *Clk* hairpin 31,661 in males) led to lifespan reduction, and the other cases showed no effect (Figure 4, Table S4). Differences in gene dosage effects together with gender‐specific attributes may have influenced the outcomes. Overall, we observed no consistent effects of manipulation of candidate genes with longevity. The data appear to suggest that the global regulatory programs, rather than changes in the expression of individual genes representing the longevity signature, are responsible for lifespan determination. However, since the transcriptome data capture only a snapshot of the cross‐species gene expression variations which are also influenced by the evolutionary history and environmental factors, further experiments will be needed to validate our findings.

## 3 CONCLUSIONS

Fruit flies have contributed significantly to our understanding of genetics and developmental biology and remain a vital tool in the studies on aging and longevity. While most experiments in the aging field have been conducted using single species, we hypothesized that the comparative analysis of closely related species with varying natural lifespan may offer unbiased information on longevity mechanisms. By studying life‐history traits and transcriptomes of 14 *Drosophila* species, we identified the pathways that diverge across these species and confirmed the role of stabilizing selection in influencing their expression patterns. By identifying the genes that correlate with longevity across these species, we found that longer‐lived species upregulate genes involved in lipid metabolism and downregulate those involved in neuronal system development and activin signaling. The dynamics of RNA polyadenylation and splicing also differed across the species.

We noted that none of the genes reached statistical significance after multiple testing correction in phylogenetic regression, suggesting that the expression patterns of many genes may not vary in a longevity‐dependent manner. This is perhaps expected, as longevity is only one of the aspects underlying the gene expression differences across these species. The statistical and predictive power was also constrained by the relatively small number of species in the current study. Furthermore, since the transcriptomic data were based on the whole body instead of specific tissues, the signals might be diluted due to gene expression heterogeneity across different tissues. Nevertheless, several enriched pathways showed FDR < 0.05 (Table S3) and the top hits were significantly enriched in protein–protein interaction (FDR < 0.001, Figure 3), suggesting that even though the genes may not reach statistical significance individually, they can still act together and produce significant changes in gene expression patterns.

For those genes with significant correlation to longevity, do they have the potential to influence lifespan individually or serve as biomarkers of longevity as a group? It appears that while some of these genes might have causative link to longevity, many others were likely mere “passengers” (their correlation might be the result, and not the cause, of longevity variation). The comparison of our results with previously reported longevity patterns produced some interesting results. On the whole transcriptome level, about two‐thirds of the genes in our cross‐species longevity signature overlapped with the expression changes observed in dietary restriction and other lifespan perturbation experiments in flies (Table 1). However, on the individual gene level, we did not observe significant overlap with the genes previously shown to increase lifespan in other model organisms (Table 2). The results of the *in vivo* shRNA knockdown analysis suggested many of the hairpins did not affect lifespan or only showed the effect in one gender. Gene dosage may have played a role too, for example, some genes previously reported to influence lifespan [e.g., *Cdk5* in flies (Connell‐Crowley et al., 2007) and *Clock* (ortholog of *Clk*) in mice (Dubrovsky, Samsa & Kondratov, 2010)] did not show the same effect upon shRNA knockdown. The longevity effects we observed for *Gdi* and *rab5* were consistent in both genders and agree with the prior findings in worms (Samuelson, Carr & Ruvkun, 2007; Tacutu et al., 2012). Also, *tkv* and *PH4alphaEFB* emerged as two candidates for lifespan extension in female flies (Figure 4).

The lack of consistent effects of knockdown on lifespan may be due to a number of reasons. Since the cross‐species gene expression is also influenced by evolutionary history and environmental factors, and our transcriptome data were collected from young healthy flies, the cross‐species variation we observed may be confounded by differences in physiology, metabolism, and reproduction. As the lifespan of different *Drosophila* species may have different sensitivity to temperature, this may introduce further variation in the data. Indeed, several longevity studies on *D. melanogaster* at 25°C reported mean lifespans that were longer than we observed here: 42 days (Simon, Shih, Mack & Benzer, 2003); 55–65 days (Hercus, Loeschcke & Rattan, 2003); 59–60 days (Promislow & Haselkorn, 2002); 27–36 days on baker's yeast; and 71 days on brewer's yeast (Bass, Weinkove, Houthoofd, Gems & Partridge, 2007). This suggests that other factors such as diet might also have impacted our measured lifespans.

The transcriptome provides only a snapshot of the expression landscape during early adulthood, so it may not completely reflect the dynamics of gene expression across the full lifespan of the species. Furthermore, given the high expression heterogeneity across different tissues, our whole‐body transcriptomics may be less powerful in identifying the true longevity determinants due to the weaken signals. Similarly, our whole‐body knockdown strategy (instead of a tissue‐specific knockdown) could have masked some of true effects of candidate genes, and our UAS/GAL4 RNAi system faces some technical drawbacks such as driver leakage, mosaicisms across organs and tissues, threshold effects of essential genes, and importantly, off‐target activity. Further validation will be needed to substantiate our findings here.

Overall, the results suggest that the natural variation of lifespan across closely related species may share some signatures with lifespan modulation by dietary restriction or other interventions. Although the individual candidate genes were impacted by low statistical power and relatively high numbers of false positives, they can act together to produce significant lifespan‐associated expression patterns. It appears that lifespan regulation may depend more on the overall state of the cell as represented by its gene expression, rather than on perturbation of individual genes that correlate with lifespan. Our data may serve as a starting point for further experimental analysis of gene expression states and longevity interventions that mimic natural changes in lifespan.

## 4 EXPERIMENTAL PROCEDURES

### 4.1 Fly stocks and husbandry

The 14 Drosophila species *D. ananassae* (stock number 14024–0371.13), *D. austrosaltans* (14045–0881.00), *D. biarmipes* (14023–0361.09), *D. bipectinate* (14024–0381.19), *D. erecta* (14021–0224.01), *D. kikkawai* (14028–0561.14), *D. melanogaster* (14021–0231.36), *D. mojavensis* (15081–1352.22), *D. saltans* (14045–0911.00), *D. sechellia* (14021–0248.25), *D. simulans* (14021–0251.194), *D. virilis* (15010–1051.87), *D. willistoni* (14030–0811.24), and *D. yakuba* (14021–0261.01) were purchased from UC San Diego Stock Center (La Jolla, CA, USA; Table S1). The flies were maintained on corn meal food [85.7 g corn meal “Aunt Jemima” (The Quaker Oats Company, Chicago, IL, USA), 50 ml golden A unsulfured molasses (Groeb Farms Inc, Onsted, MI, USA), 71.4 g Torula yeast (MP Biomedicals, Solon, OH, USA), 2.86 g p‐hydroxybenzoic acid methyl ester (Sigma), 6.4 g agar (MoorAgar Inc, Loomis, CA, USA), and 5.7 ml propionic acid (Sigma) per liter water] and kept in a temperature‐controlled incubator at 25°C with 12‐h light/dark cycle and ~60% humidity. These species were selected because they could grow and reproduce well on the sugar‐yeast diet.

For the lifespan assays, the newly emerged male and virgin female flies were collected within 18 h at 18°C (lower temperature to reduce first mating) and transferred to fresh corn meal food at density of 35 animals per vial for 2–3 days at 25°C. The flies were then allowed to mate for 1–2 days, collected using CO_2_, sorted by sex, and transferred to the specially designed cages in temperature‐controlled incubator at 25°C (Figure S1). The experimental flies were held on the designed diet and transferred to fresh food vials without anesthesia every 3 days. Dead flies were removed by aspiration and counted. Three biological replicate cages were used per gender per species, and the detailed life tables are shown in Table S1. Survival analyses were performed using R package “survival” and Kaplan–Meier method (Kaplan & Meier, 1958; Therneau, 2014).

### 4.2 RNA sequencing

Since the gene expression patterns in female flies may have greater variation during egg‐laying and reproduction, we selected male flies for RNA‐seq analysis. Three‐day‐old male flies were placed in vials on the corn meal diet for 12 days, with three replica vials for each species. Fresh food was supplied every 3 days, and dead flies were removed by aspiration. After 12 days, the flies were subject to total RNA extraction. The RNA sequencing libraries were constructed using the Illumina mRNA‐Seq Prep Kit, and oligo(dT) magnetic beads were used to purify polyA containing mRNA molecules. The mRNA was further fragmented and randomly primed during the first strand synthesis by reverse transcription, and then by second‐strand synthesis with DNA polymerase I to create double‐stranded cDNA fragments. The double‐stranded cDNA was end‐repaired by Klenow and T4 DNA polymerases and A‐tailed by Klenow lacking exonuclease activity. This was followed by ligation to Illumina paired‐end sequencing adapters, size selection by gel electrophoresis, and PCR amplification. The 200‐bp paired‐end libraries were sequenced using Illumina HiSeq according to the manufacturer's protocol. The raw reads are deposited in Sequence Read Archive (SRA), accession number SRP119808, BioProject PRJNA414017.

### 4.3 Ortholog set identification

The ortholog sets across the species were identified by reciprocal best hits in BLAST. Briefly, we downloaded the genomes and annotation files for the species from Ensembl and NCBI (Table S1) and extracted all the coding sequences (“Species CDS”). The genomes of *D. austrosaltans* and *D. saltans* were based on our unpublished data. As reference, we extracted the longest open‐reading frame for each gene in *D. melanogaster* (“Dmel ORF”), after excluding those with multiple paralogs (i.e., genes with over 80% identity over 70% of length) or highly repetitive sequences. Mega BLAST (Morgulis et al., 2008) was performed to obtain reciprocal best hits between Dmel ORF and Species CDS. An ortholog set was declared if the orthologs were present in all 14 species. We confirmed our list had over 90% overlap with the curated ortholog list on Flybase (St Pierre et al., 2014; which covered nine of our species). To improve the quality of ortholog sequences, Trinity (Grabherr et al., 2011) was also used to *de novo* assemble the transcriptomes from the RNA‐seq data (Table S1). Poorly annotated Species CDS (e.g., those without proper start or stop codons) were replaced by Trinity (Grabherr et al., 2011) transcripts where applicable, and we ensured that the final list of orthologs contained at least 20% conserved blocks in the multiple sequence alignment. The final ortholog sequences were mapped back to their respective genomes with GMAP (Wu & Watanabe, 2005) to generate customized GFF (General Feature Format) files.

### 4.4 Data processing

RNA‐seq reads alignment was performed using TopHat (Trapnell, Pachter & Salzberg, 2009), and read counting was performed using featureCounts (Liao, Smyth & Shi, 2014). Those ortholog sets with low expression (i.e., <3 counts in three or more species) were removed, and the counts were normalized by total library sizes with trimmed mean of M‐values correction (Robinson, McCarthy & Smyth, 2010; Robinson & Oshlack, 2010). The final list consisted of 6,510 ortholog sets. The normalized counts were then converted to reads per kilobase per million mapped reads (RPKM) values and natural log‐transformed. For cross‐species comparison, log‐RPKM values of each ortholog set were standardized by setting mean as 0 and standard deviation as 1. Q‐Q plot and Shapiro–Wilk test confirmed that normalcy was a valid assumption for 88% of the ortholog sets on log‐scale. The read counts and log‐RPKM values are found in Dataset S1.

We compared our expression data with two previous studies of gene expression across multiple *Drosophila* species (Chen et al., 2014; Zhang et al., 2007)—GSE99574 is based on RNA‐seq and GSE6640 is based on microarrays (herein referred to as “reference studies”). For simplicity, we used only “male replicate 1” in each reference study for whole‐body expression. The following steps were applied to each reference study: (i) extract the species and the gene orthologs common to our study and the reference study; (ii) quantile normalization; (iii) for each gene, rank the expression across the samples within our study and within the reference study; (iv) compute a pairwise distance matrix of average absolute rank differences for all sample pairs; (v) perform hierarchical clustering using average linkage.

### 4.5 Divergence time and phylogram

The *Drosophila* species divergence time was estimated based on previous estimates (Russo et al., 2013) and the ortholog amino acid sequences. Briefly, the ortholog sequences were aligned with Clustal Omega v1.2.0 (Sievers et al., 2011) and concatenated gap‐free with Gblocks v0.91 (Castresana, 2000). The tree was constructed using neighbor‐joining method (Saitou & Nei, 1987) in Mega 6.06 (Tamura, Stecher, Peterson, Filipski & Kumar, 2013) and calibrated using the estimates of divergence time in the literature. The *Drosophila* expression phylogram was based on a distance matrix of 1 minus Spearman correlation coefficient and constructed by neighbor‐joining method using R package “ape” (Paradis, Claude & Strimmer, 2004). Reliability of the branching pattern was assessed by a 1,000‐time bootstrap across the genes, using “boot.phylo” function of R package “ape” and random sampling of the expression set with replacement. For the mammalian dataset (Brawand et al., 2011), the amino acid sequence alignments of eight mammalian species (human, gorilla, chimpanzee, orangutan, macaque, mouse, opossum, and platypus) were extracted from the 46‐way multiple alignment in UCSC genome browser (Kuhn, Haussler & Kent, 2013). The species divergence time was based on TimeTree database (Hedges, Dudley & Kumar, 2006).

### 4.6 Expression divergence

The expression divergence was measured as average expression variance in the standardized expression values across all the ortholog sets between the species pairs. The points were fitted by the model previously described (Bedford & Hartl, 2009):

$$y\;=\;\frac{\sigma^{2}}{2\alpha }\left(1-e^{-2\alpha x}\right)$$

where *x* represents the divergence time, *y* represents the expression divergence, α represents the strength of selection, and σ^2^ represents the strength of drift. It can be shown that (in the case of a pure BM model):

$$\begin{array}{c} \lim\\ \alpha \to 0 \end{array}\;y\;=\;\sigma^{2}x$$

Optimal values of the parameters were estimated by least‐squares method. Confidence intervals (C.I.) were estimated by a 1,000‐time bootstrap, where the gene sets were sampled with replacement. To test the robustness of the relationship, individual species were removed one at a time and the remaining species were subjected to the same analysis. The minimum and maximum values obtained in this procedure were then reported.

### 4.7 Principal component analysis and pathway enrichment

Principal component analysis (PCA) was performed on the standardized expression values using R package “stats” (R Development Core Team 2013). To identify the underlying pathways, the genes in each of the first three principal components (PCs) were ranked by their contributions and pathway enrichment analysis was performed on the top 300 (about 5%) genes in each PC using DAVID (Huang da, Sherman & Lempicki, 2009; Huang da, Sherman & Lempicki, 2009) after correcting for background. To generate the heat map, the genes (columns) were ordered by contributions and the species (rows) were ordered by projection values.

### 4.8 Mode of evolution of individual genes

The phylogenetic signals (Pagel's λ and Blomberg's K) were calculated using R package “phytools” (Revell, 2012). To model the gene evolution, individual genes were fitted to a BM model (null hypothesis) and an OU model with one to three optima (alternative hypothesis), using R package “ouch” (King & Butler, 2009). The optima for OU model were chosen based on taxonomical grouping. The goodness of fit of each OU model was compared against BM model, using likelihood ratio test (Butler & King, 2004).

### 4.9 Regression by generalized least square

Regression was performed by generalized least‐squares method using R packages “nlme” and “phylolm” (Ho & Ane, 2013; Pinheiro, Bates, DebRoy & Sarkar, 2013). The trait data were log‐transformed and standardized. Four models of trait evolution were tested: (i) complete absence of phylogenetic relationship (“Null”); (ii) Brownian motion model (“BM”); (iii) BM transformed by Pagel's lambda (“Lambda”); and (iv) Ornstein–Uhlenbeck model (“OU”; Felsenstein, 1985; Lavin, Karasov, Ives, Middleton & Garland, 2008; Ma et al., 2015; Martins & Hansen, 1997; Pagel, 1999). These models describe the relationship among the regression residuals of the species. Briefly, the null model assumes 0 covariance between the species (i.e., independent evolutionary trajectory). The BM model assumes the amount of changes in a trait is proportional to time, and the residual covariance between a pair of species is proportional to the shared branch lengths (i.e., shared evolutionary trajectory). Both the Lambda and OU models are variations of the BM model: The Lambda model scales the covariance by a factor lambda ranging between 0 and 1, where the OU model includes a selection strength parameter and an optimal trait value, such that the trait values of the species will be pulled toward the optimal trait value. For the Lambda and OU models, the parameters were estimated simultaneously with the coefficients using maximum likelihood. The best‐fit model was selected based on maximum likelihood. The strength of correlation was based on the *p*‐value of regression slope.

### 4.10 Comparison with GenAge/GenDR database and microarray data

Our gene list was examined against GenAge/GenDR databases (de Magalhães et al., 2009; Plank et al., 2012) to determine how the longevity effects reported in model organisms relate to our gene dataset. To analyze microarray datasets, data were downloaded from Gene Expression Omnibus (GEO) database. We used the search term “Drosophila melanogaster AND dietary restriction” and study type “Expression profiling by array” to identify nine datasets initially. Among them, we excluded three time‐course studies, one study focusing on expression in fat body, and one study where the RNA was preprocessed by sucrose gradient separation. For the remaining datasets (GSE37537, GSE26724, GSE48145, GSE26726), relevant comparisons of treatment versus control were selected and differentially expressed (DE) genes were identified using R package “limma” (Smyth, 2005). These DE genes were then compared with our list of top hits to determine if the direction of correlation was consistent.

Two methods were employed to calculate *p*‐values. The first relied on binomial distribution, counting the number of match (i.e. same direction of correlation) and the number of mismatch (i.e., opposite direction of correlation), assuming equal probability of obtaining a match and a mismatch by chance. The second method relied on simulation, where the direction of correlation in our top list was sampled without replacement and compared with microarray experiments to calculate *p*‐values (by binomial distribution); this procedure was repeated 1,000 times to generate an empirical distribution. The original *p*‐value was then compared to the empirical distribution. Both methods produced very similar results.

### 4.11 Gene function studies and lifespan analysis

RNAi studies were carried using GeneSwitch (GS) Gal4 System (Huang, Lu‐Bo & Haddad, 2014; Nicholson et al., 2008; Roman, Endo, Zong & Davis, 2001) and transgenic shRNA lines from Transgenic RNAi Resource Project (TRiP). The actin‐GS‐Gal4 virgin females were crossed to respective transgenic shRNA lines. For the lifespan analysis, we maintained the flies in vials to allow for a quicker turnaround of counts and food changes on large groups. The remaining protocols, including husbandry, mortality counts, and food changes, were similar as described above. Briefly, *D. melanogaster* males and females were collected for 3 days and allowed to mate for another 2–3 days. These flies were then placed into vials at relaxed density (20–30 individuals per vial) and allowed to age at 25°C. Food changes were performed every 3 days, and dead flies were counted and removed. Transcription of the transgenic shRNA was induced by RU486 (150 μg/ml). Three biological replicates were used for each gender (male vs. female) and treatment (RU486‐treated vs. control). Kaplan–Meier statistics and hazard ratio were calculated using R packages “survival” and “survcomp” (Schroder, Culhane, Quackenbush & Haibe‐Kains, 2011; Therneau, 2014). A treatment was considered significant if both the G‐rho family tests *p*‐value < .01 and the hazard ratio > 1.5.

## CONFLICT OF INTEREST

None declared.
