# Supplementary material for: Comparative transcriptomics across 14 Drosophila species reveals signatures of longevity
Source: Aging Cell. 2018 Apr 19;17(4):e12740. doi: 10.1111/acel.12740 (PMC6052463; doi:10.1111/acel.12740)
Supplement: Supplementary file 6 [file ACEL-17-na-s006.pdf]

Supplementary Information

Table S1. Species information, RNAseq, and life history.

Table S2. Pathway enrichment analysis of top 300 genes in the first three Principle Components. Enrichment analysis was performed using DAVID.

Table S3. Genes with significant correlation to median lifespan (ML). “Best.Model” indicates the best-fit regression model; “Benjamini” indicates the p-value adjustment for multiple testing (Benjamini & Hochberg 1995); “p.val (excl D.virilis)” indicates the p-values excluding the *D. virilis* data. Enrichment analysis was performed using DAVID, separately for genes with positive and negative correlations.

Table S4. Longevity effects of shRNA knockdown of selected genes. The mean (“Mean”) and standard error (“SE”) of lifespan and the number of flies (“N”) are indicated. “Coef” refers to the coefficient of the Cox Proportional-Hazards Model, and the hazard ratio (“hazard”) is calculated as exponent of the absolute coefficient. “p-value” refers to the G-rho family tests p-value. The directions of lifespan changes are indicated for those hairpins with significant longevity effects (p-value < 0.01 and hazard > 1.5).

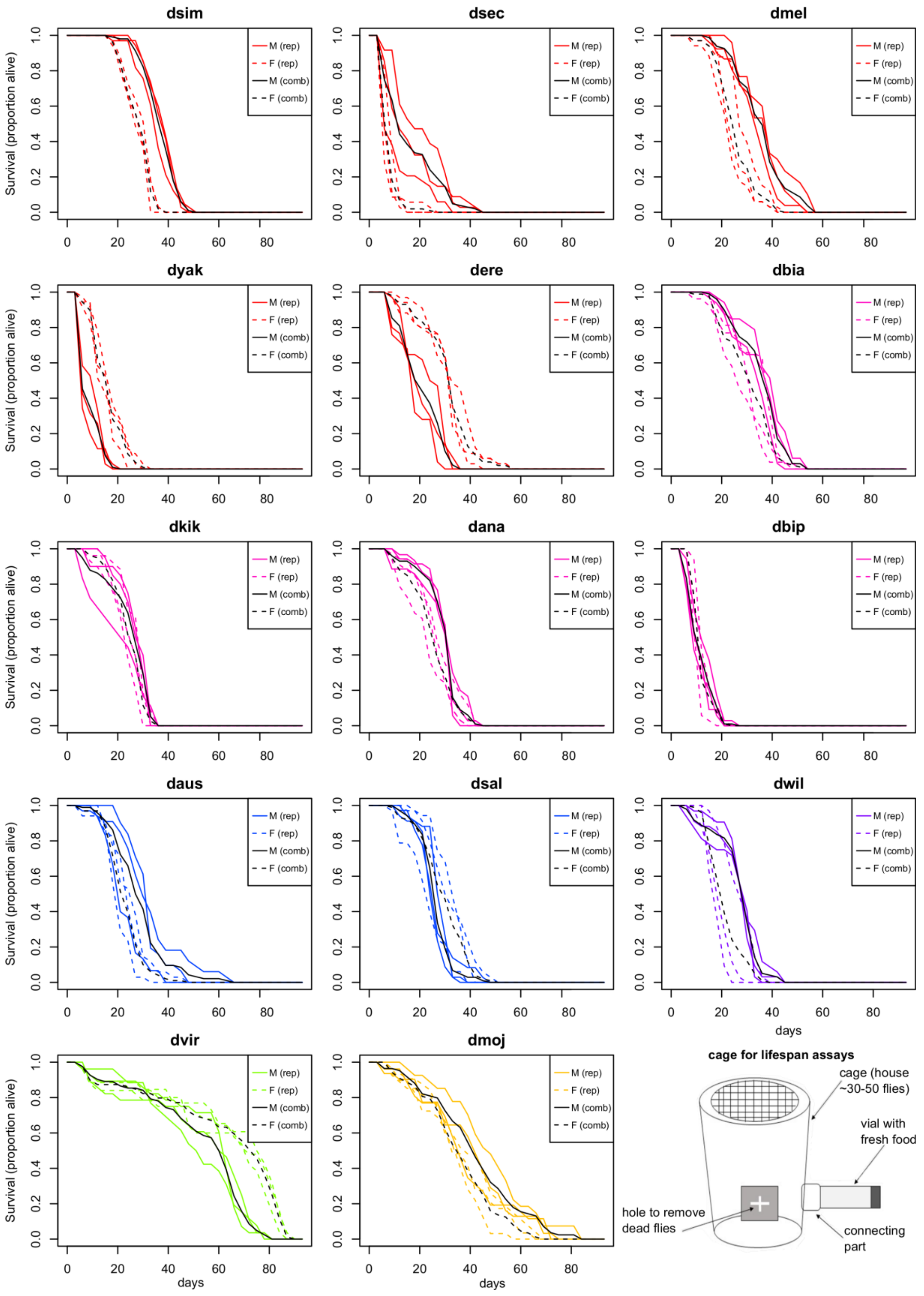

Figure S1. Survival plots for the species in this study. Survival curves for 3 male replicates ["M (rep)"] and 3 female replicates ["F (rep)"], as well as the combined data for male ["M (comb)"] and female ["F (comb)"] are shown. See also Table S1. Cage design for lifespan assays is shown in bottom right.

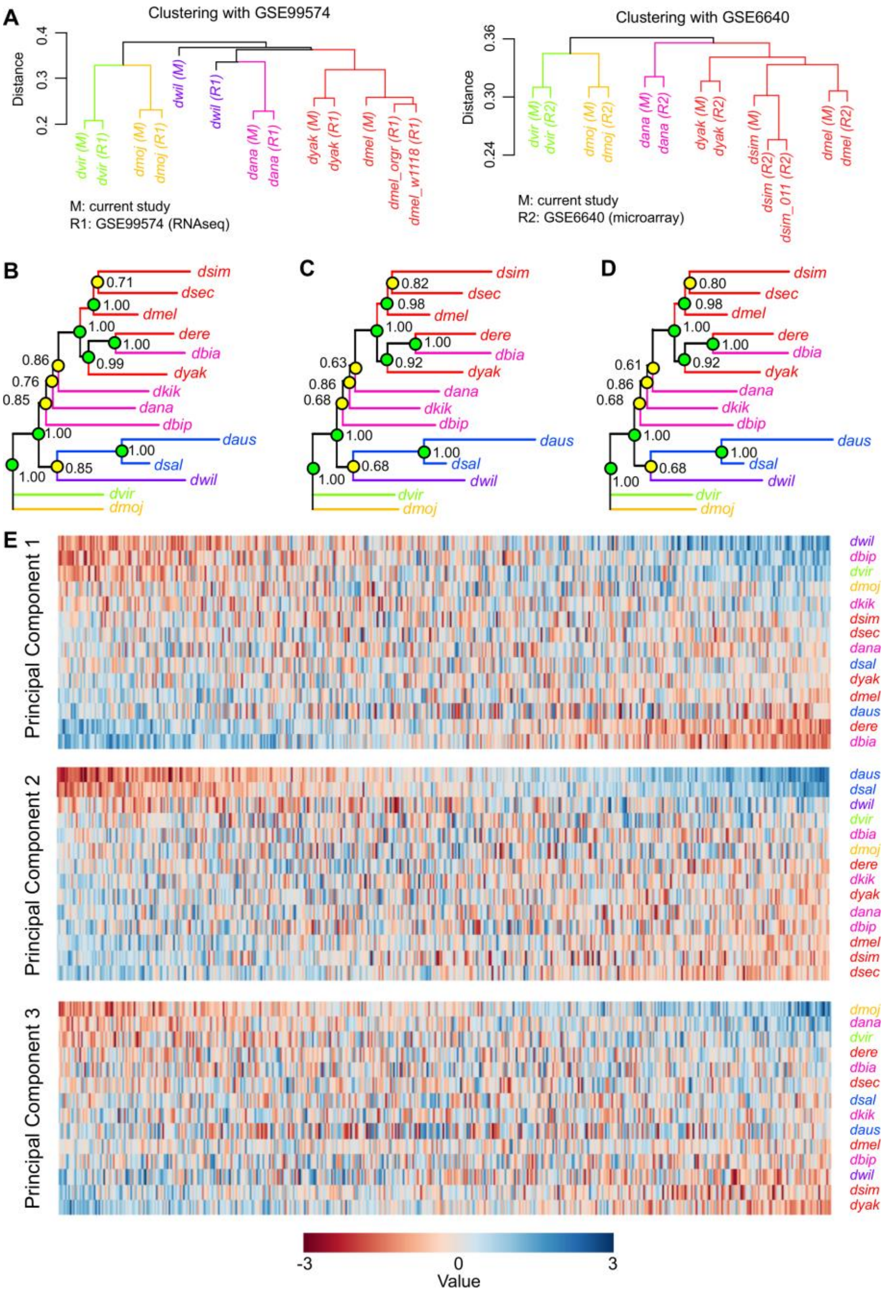

Figure S2. Gene expression across species. (A) Clustering with previous expression studies. “dmel_ongr” and “dmel_w1118” refer to two strains of *D. melanogaster* tested in GSE99574. “dsim_011” refers to *D. simulans* strain 14021-0251.011 in GSE6640. **(B) – (D) Gene expression phylograms constructed by alternative methods.** The distance matrix was based on (B) 1 minus Pearson correlation coefficient; (C) mean squared differences; or (D) mean variance. The reliability of the branching pattern was assessed by 1000-time bootstrap across the genes (bootstrap values next to the nodes; green: ≥0.9; yellow: 0.6-0.9). **(E) Heat maps showing contribution of genes to the first three Principal Components (PC).** In each heat map, the genes (columns) are ordered by their contributions to each PC and the species (rows) are ordered by their projection values on each PC.

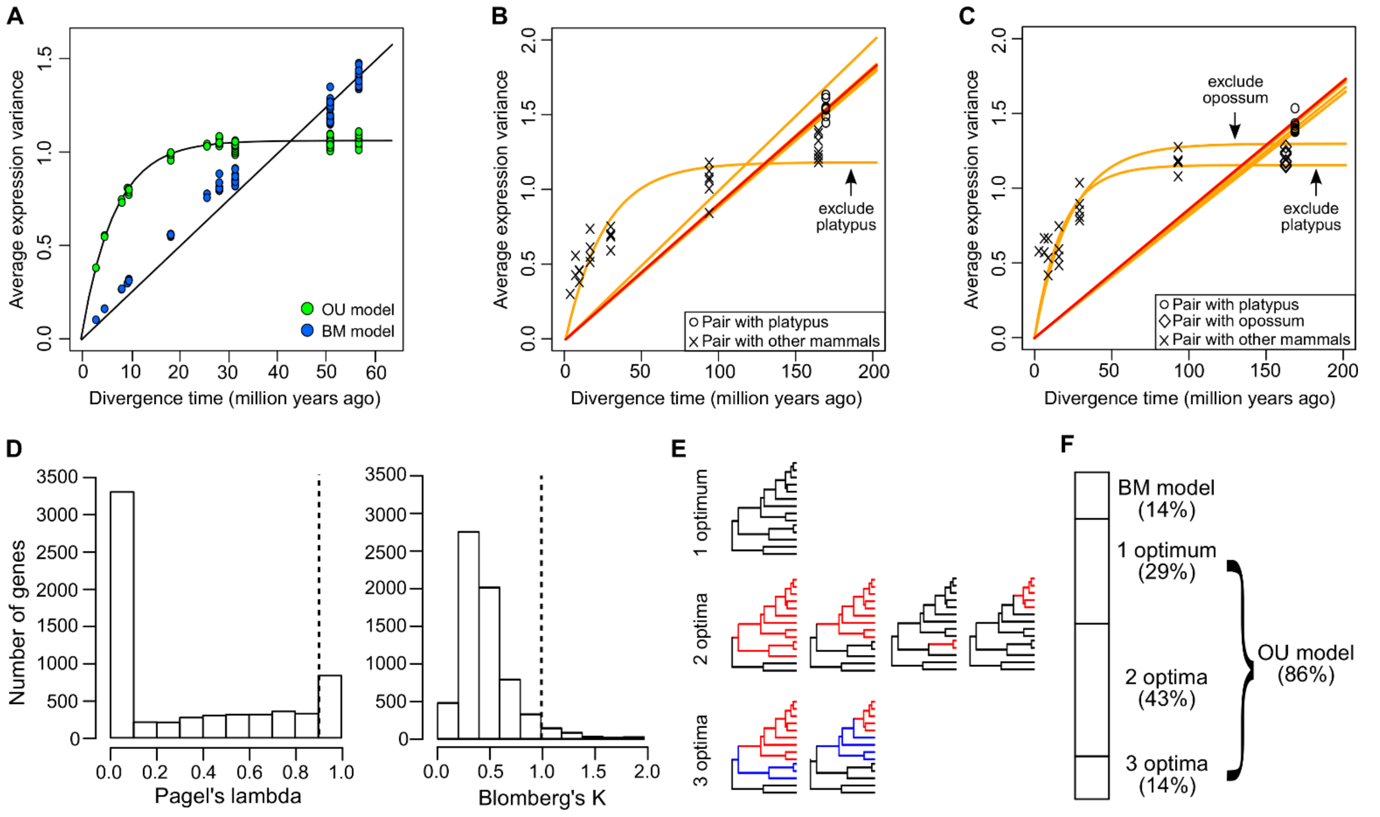

Figure S3. Expression divergence and evolutionary model. (A) Simulation under Orstein-Uhlenbeck (OU) and Brownian Motion (BM) models. Data were simulated for 6000 genes using the phylogenetic tree in Fig. 1, as well as the α (for OU only) and σ^2 (for both BM and OU) values estimated in Fig. 2C. **Average expression variance in (B) brain and (C) liver of 9 mammalian species.** The species are human, gorilla, bonobo, chimpanzee, orangutan, macaque, mouse, opossum, and platypus. Estimated parameters: (B) $\alpha<10^{-5}$ (95% C.I.: 10^{-5}–10^{-5}), σ^2=0.00916 (95% C.I.: 0.00911–0.00922); (C) $\alpha<10^{-5}$ (95% C.I.: 10^{-5}–10^{-5}), σ^2=0.00866 (95% C.I.: 0.00861–0.00873). Red lines indicate the best-fit lines using all the species. Orange lines indicate the best-fit lines when one of the species is excluded. **(D) Pagel's λ and Blomberg's K were small in many genes.** Right side of the dotted lines indicates the genes with strong phylogenetic signals (i.e. Pagel's $\lambda > 0.9$ or Blomberg's $K > 1$). **(E)** Gene expression fitted by OU models with up to 3 optima. The phylogenetic tree is the same as Fig. 1, with the tip labels omitted. **(F)** Percentage of genes best fitted by each of the models. The goodness of fit of the models was determined by likelihood ratio test.

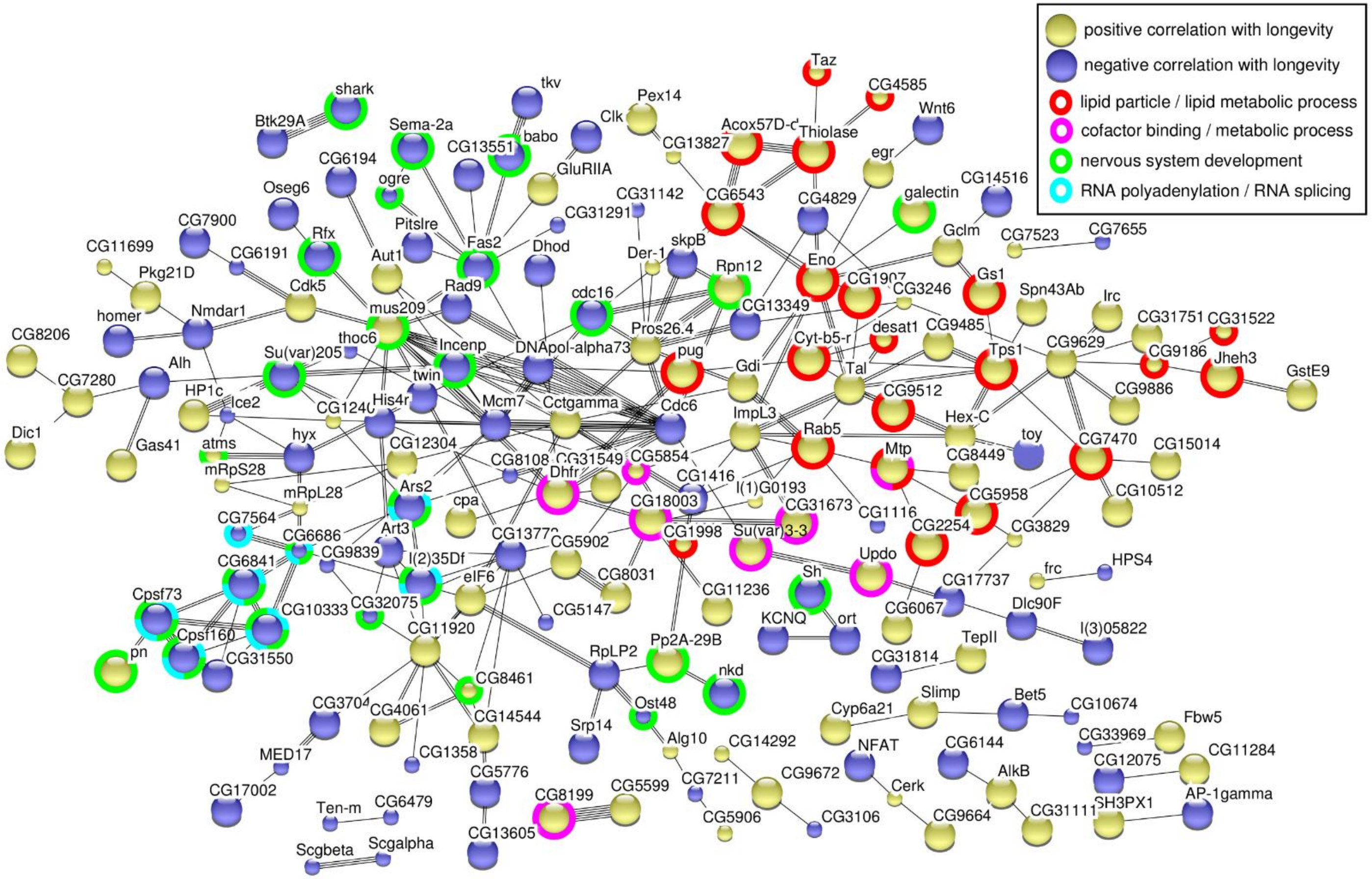

Figure S4. Protein-protein interaction network among the top hits correlating with median lifespan. The interaction network was based on STRING database (evidence view). Genes without interacting partners are omitted.
